# Antigen-Specifically Activated CD8+ and Double-Negative T Cells Accumulate in the Brain of Alzheimer’s Disease Mice

**DOI:** 10.14336/AD.2025.0452

**Published:** 2026-06-27

**Authors:** Juliane Gellrich, Johanna Ruhnau, Lea Köslich, Stefan Gross, Agnes Flöel, Juliane Schulze, Antje Vogelgesang

**Affiliations:** ^1^Department of Neurology, University Medicine, 17475 Greifswald, Germany.; ^2^Department of Inner Medicine B, University Medicine, 17475 Greifswald, Germany.

**Keywords:** Alzheimer’s disease, T cell activation, DNT, CD8+ T cells, antigen-specific

## Abstract

Emerging evidence suggests adaptive immunity plays a key role in cognitive function and neurodegenerative diseases. However, the specific contribution of T cells in Alzheimer’s disease (AD) remains poorly understood. Despite successful T cell modulation in other neurological conditions, similar strategies in AD remain underexplored due to gaps in our understanding of antigen-specific T cell activity and antigen-unspecific bystander activation in the diseased brain. In this study, we used flow cytometry to characterize T cell populations and their activation mode in an AD mouse model. By assessing GFP expression in C57BL/6J-Tg(Nr4a1-EGFP/cre)820Khog; Tg(APPswe,PSEN1dE9)85Dbo/Mmjax mice, we distinguished antigen-dependent from antigen-independent activation in CD4^+^, CD8^+^, and double-negative T cells (DNTs). This approach allows analysis of the full repertoire of antigen-specifically activated T cells in a physiological immune system without prior knowledge of target antigens. AD-like amyloid pathology progression was monitored by monthly scoring until mice reached 2, 6, 10-12 or 15-18 months of age and Aβ-quantification via thioflavine S staining. Antigen-specific activation during AD development was assessed by comparing AD mice with wild-type littermates. At 15-18 months, AD mice exhibited elevated numbers of activated, highly differentiated DNTs, along with increased antigen-specific CD8^+^ and DNT cells relative to controls. These results indicate a significant role for antigen-dependent immune activity in AD, highlighting CD8^+^ T cells and DNTs as potential therapeutic targets

## INTRODUCTION

Dementia affects over 55 million people worldwide and a drastic increase to 139 million people is expected within the next 25 years, causing a continuously growing socioeconomic burden [1]. Alzheimer's disease (AD), the most common form of dementia, is an age-related neurodegenerative disorder characterized by gradual cognitive decline, primarily involving memory loss [1, 2]. Accumulation of extracellular amyloid β (Aβ) plaques and intracellular neurofibrillary tangles (NFT) are characteristic hallmarks of AD, but the complex pathomechanism comprises additional facets like vascular dysfunction and (neuro-)inflammation [2].

Systemic inflammation — triggered by infectious diseases as well as non-infections conditions like atherosclerosis, obesity or diabetes — has emerged as a significant contributor to the pathogenesis of AD. Chronic peripheral inflammatory conditions are associated with a heightened risk for developing AD and in preclinical studies, systemic inflammation has been shown to accelerate and enhance Aβ plaque and NFT deposition [3-8]. In AD, elevated proinflammatory cytokines upregulate adhesion molecules and disrupt the blood-brain and blood-CSF barriers, allowing peripheral immune cells and inflammatory mediators to access the brain parenchyma [9, 10]. Once in the brain, these factors can exacerbate microglial activation, promote amyloid-beta deposition, and accelerate neuronal injury [10, 11]. This crosstalk between peripheral and central immune responses underscores the role of a general systemic inflammation as both a potential trigger and amplifier of AD pathology, highlighting the need to consider comorbid inflammatory diseases in the broader understanding and treatment of Alzheimer’s disease. In addition to general inflammation that can accelerate AD pathology, specific immune responses have also been shown to play a critical role in the progression of the disease. There is substantial evidence indicating that T cells are involved in AD pathology. The activation of T cells is elevated [12-15], and T cells accumulate within the AD brains [16-21]. Aβ-specific T cell reactivity aids in amyloid clearance [22] and is increased in both AD patients and healthy elderly controls compared to young controls [23]. However, AD patients show a decreased antigen-specific T cell response towards Aβ as well as mitochondrial and microbial peptides compared to age-matched controls that might be caused by T cell anergy, suggesting functional impairment with a reduced ability to remove harmful metabolic products [24, 25].

The role of T cells in disease progression varies between different transgenic mouse models, emphasizing the need for further characterization of specific T cell responses.

T cells are a heterogeneous population of CD4+, CD8+ and double negative (CD4- CD8-) cells essential for the immune system, especially in coordinating responses and maintaining immune balance. Among them, CD4+ T helper (Th) cells are key regulators and can differentiate into distinct subgroups based on the signals they receive. The main T helper subgroups include the proinflammatory Th1 and Th17, the anti-inflammatory Th2, and regulatory T cells, each with unique functions and cytokine profiles [26]. T cells may be beneficial or detrimental in AD, depending on timing and their functional subgroup: Adoptive transfer of Aβ-specific T cells in a mouse model of AD reversed cognitive deficits and Th2 and Treg cells have been identified as important protective subsets [27-29]. Th1 and Th17 cells activated microglia, which was protective in some [30, 31] and detrimental in other studies [28, 32].

CD8+ T cells represent the majority of T cells in the AD brain, are elevated in the blood and cerebrospinal fluid of AD patients and contribute to lesion development [15, 16, 18, 19, 33, 34]. However, recent studies suggest a protective role for CD8+ T cells, as lower CD8+ counts and the elimination of brain-infiltrating CD8+ T cells have been linked to increased amyloid-beta deposition and worsened cognitive impairment [18, 35]. Additionally, peripheral double-negative T cells (CD4-/CD8-, DNT) are elevated in AD and have been shown to worsen AD pathology by amplifying the proinflammatory environment [36-40].

Treatment options for AD remain limited. Current research focuses on improving antibody therapies by addressing immunosenescence and incorporating therapeutic T cell strategies [41-43]. Immunosenescence and immune exhaustion are also targeted by PD-L1-blockade, which is currently tested in patients with early AD (NCT05551741). So far, our understanding of the role of T cells in AD remains insufficient to develop targeted therapies. Determining the precise functions, antigen-specificity, timing, and influence of different T cell subsets in AD progression will advance the development of targeted immunomodulatory treatments.

This study utilized the APP/PS1 mouse model of Alzheimer's disease in combination with the Nur77^GFP^ mouse model to explore the mode of activation in different T cell subpopulations including CD4+, CD8+ and DNT during different stages of AD. We examined whether T cells are activated through their T cell receptor (TcR), suggesting an antigen-specific and potentially autoimmune role, or through a bystander activation via the proinflammatory environment without TcR involvement. While it is known from other studies that antigen-specific immune responses are of importance in AD pathology [44, 45], our model enables investigation of antigen-specifically and antigen-unspecifically activated T cells side by side within a physiological immune system during the course of disease progression to report the extent of both activation modes simultaneously within the brain. Although some antigens relevant in AD development have already been identified, others have most likely been overlooked. Our approach enables investigation of the whole antigen-specifically activated T cell repertoire. Understanding the differences between antigen-specific and -unspecific T cell activation will support the development of immunomodulatory therapies targeting distinct T cell subsets or antigens.

## MATERIALS AND METHODS

### Animals and housing

All animal experiments and the calculated sample sizes were approved by the local government authorities (Landesamt für Landwirtschaft, Lebensmittelsicherheit und Fischerei (LALLF) Mecklenburg-Vorpommern, 7221.3-1-006/22) and comply with the ARRIVE guidelines as well as the EU Directive 2010/63/EU for animal experiments.

We utilized a transgenic mouse model to identify antigen-specifically activated T cells via green fluorescent protein (GFP) expression in mice developing AD-like amyloid pathology. The Nur77^GFP^ mouse model was generated by Kristin Hogquist on a C57BL/6 background (C57BL/6-Tg(Nr4a1-EGFP/cre)820Khog/J mice, Stock No: 016617, The Jackson Laboratory) [46]. Nur77^GFP^ mice express eGFP under the control of the Nr4a1 (Nur77) promoter/enhancer regions. Nr4a1 encodes the immediate early gene Nur77, which is expressed after T cell activation via antigen receptor stimulation but not by inflammatory stimuli alone. GFP is expressed transiently (for about 72h), reaching its maximum approximately 24h after TCR stimulation. [46]

Nur77^GFP^ mice were bred with APP/PS1 mice (B6.CgTg(APPswe,PSEN1dE9)85Dbo/Mmjax) (Jackson Laboratory) that develop symptoms of Alzheimer's disease (C57BL/6J-Tg(Nr4a1-EGFP/cre)820Khog; Tg (APPswe,PSEN1dE9)85Dbo/Mmjax). APP/PS1 mice express a chimeric mouse/human amyloid precursor protein (Mo/HuAPP695swe) and mutated human presenilin 1 (PS1-dE9), which affect central nervous system neurons and lead to an early onset of AD-like amyloid pathology and cognitive decline at the age of 6 months [47].

We compared mice with AD-like amyloid pathology, further named “AD” (Nur77^GFP^-APP/PS1: C57BL/6J-Tg(Nr4a1-EGFP/cre)820Khog; Tg(APPswe,PSEN1dE9) 85Dbo/Mmjax) mice to a control group, named “wt” (Nur77^GFP^:C57BL/6-Tg(Nr4a1-EGFP/cre)820Khog/J mice).

**Figure 1.**
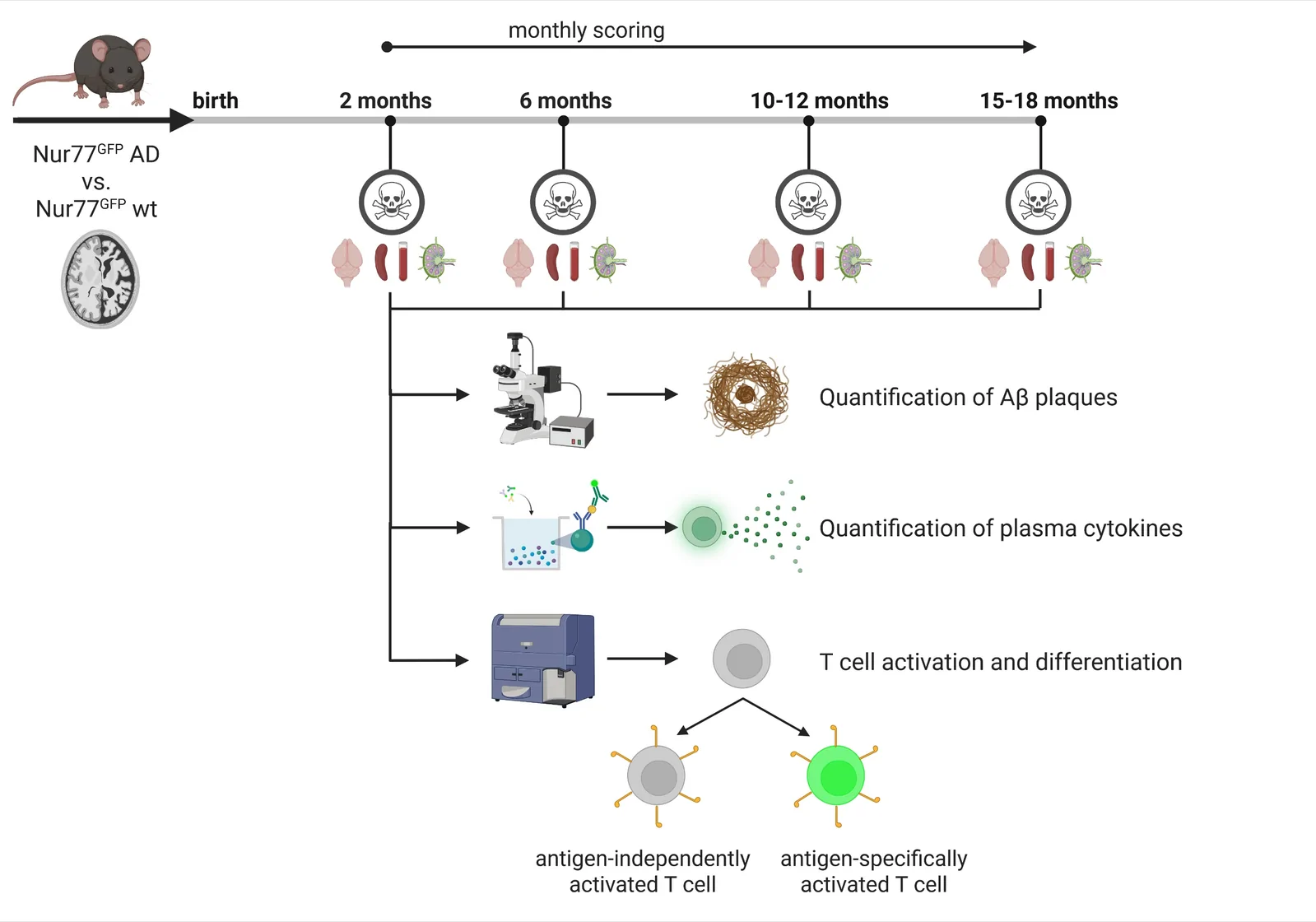
**Study design**. Alzheimer’s-Nur77 (AD) and wildtype-Nur77 (wt) mice were included in the study at the age of 2 months and scored monthly until they reached their respective experimental endpoint (2, 6, 10-12, 15-18 months). Then, brain, spleen, blood and cervical lymph nodes were collected for flow cytometric analysis of T cell activation, cytokine quantification and thioflavine S staining of amyloid β plaques. Figure created with biorender.com.

Female mice with the appropriate genotype were included at the age of 2 months and monitored monthly until they reached their respective experimental endpoint (2, 6, 10-12, 15-18 months). (Fig. 1) Since the disease course and severity differs between male and female mice [48], comparison of both sexes would increase data variability and the number of animals needed. For ethical reasons, in this first study we only investigated female mice, since AD predominantly affects women [49]. Further studies are needed to verify our results in male mice.

Researchers were blinded to the type of genotype and outcome during sample preparation and data evaluation.

Mice were bred locally at the Central Service and Research Facility of the University Medicine Greifswald and group-housed in an enriched environment (paper rolls, nesting material) under specific pathogen-free conditions. All animals were maintained under a 12 h light/dark cycle and had ad libitum access to standard chow and acidified tap water (pH=2.5). Room temperature and humidity were monitored and kept at 20 ± 2 °C and 60 ± 20 %, respectively.

### Monthly scoring

Starting at the age of 2 months, mice were scored monthly to ensure animal welfare and monitor disease development. Weight and temperature were recorded and a sum score consisting of 5 subscores (general condition, fur condition / fur care, activity, posture/movement, induced behavior), each on a scale from 0 to 3, was formed (Supplementary Table 1). No mice needed to be excluded due to reaching the humane endpoint (sum score >10 or score of 3 in one category) (Fig. 1)

### Organ harvesting

At each time point of sacrifice (2, 6, 10-12, 15-18 months), mice were deeply anesthetized with 3 % isoflurane in 1 l/min oxygen and transcardially perfused with HBSS (Hanks’ Balanced Salt Solution; w/o Ca/Mg). Transcardiac perfusion was performed in adaptation of the protocol published by Wu et al. [50]. A lateral incision was made just below the rib cage, and the liver and gallbladder were carefully separated from the diaphragm. The xiphoid process was then grasped with a large hemostat and secured over the mouse's head. Following this, the diaphragm was incised along the full length of the rib cage, and the rib cage was cut on both sides. Immediately before perfusion, blood was collected from the left ventricle by cardiac puncture (25G needle) and anticoagulated with EDTA. A 24G safety cannula (Introcan) was inserted into the left ventricle, and the needle was withdrawn, leaving only the plastic cannula in place. Cold HBSS was perfused through the cannula, and an incision was made in the right atrium to allow outflow. Perfusion was continued until the liver and paws appeared clear, typically after the passage of approximately 20-30 mL of cold HBSS. After perfusion, brain, spleen and cervical lymph nodes (CLN) were collected and maintained in HBSS (w/o Ca/Mg) until cell isolation:

First, a vertical midline incision was made, and the spleen was isolated by gently grasping the surrounding adipose tissue with forceps. Fine scissors were used to carefully detach the spleen from adjacent connective tissue.

Next, the incision was extended cranially toward the mouth, and the skin was retracted laterally to expose the submandibular glands. The superficial cervical lymph nodes—three on each side, embedded within the parotid, sublingual, and submandibular glands—were identified and extracted using fine forceps.

Finally, the skull was exposed by decapitating the mouse at the level of the first cervical vertebra, followed by a midline incision from the neck to the nose to remove the overlying skin. Caudal bones and muscles were trimmed, and the skull was carefully opened starting at the foramen magnum using fine forceps. After removing the meninges, the brain was extracted with a micro spatula by gently severing the cranial nerves.

Plasma was prepared by centrifugation of whole blood (17 800g, 5 min) and frozen at -80 °C for later analysis.

### Brain processing

The protocol for brain processing was adapted from Pösel et al. [51]. Olfactory bulbs and spinal cord were cut off and the brain (including the cerebellum) was split into two parts along the median longitudinal fissure. One half was put aside for thioflavine S staining (see below) and the other half was mechanically homogenized using a 100 µm cell stainer (Greiner bio-one) and a plunger of a 2 mL syringe, flushing with HBSS with Ca/Mg. After centrifugation (300xg, 5 min, 4°C, 7/7), the cell pellet was resuspended in 2 mL wash buffer (HBSS w/o Ca/Mg + 10 % FCS) containing DNASe I (Applichem, 668 U/mL) and flushed trough a 70 µm cell strainer using DNAse-free wash buffer. After centrifugation (300xg, 5 min, 18 °C, 7/7), the pellet was resuspended in 25 % Percoll (Percoll Plus, Sigma) for density centrifugation (500xg, 20 min, 18 °C, 9/1). Only the cell pellet at the bottom was kept and washed with HBSS (w/o Ca/Mg) (300xg, 5 min, 4°C, 7/7). Finally, cells were resuspended in 100 µL HBSS (w/o Ca/Mg) for flow cytometry staining. Spleen, CLN and blood processing are described in the supplement (Supplementary S2).

### Flow cytometry staining and analysis

100 µL cell suspension was incubated with Zombie Aqua (BioLegend, REF: 423102) for dead cell removal. After washing with fluorescence-activated cell sorting (FACS) buffer, the samples were incubated with TruStainfcX™ (anti-mouse CD16/32, clone 93, BioLegend, REF: 101320) for 10 min at 4 °C to prevent unspecific staining. Cells were subsequently stained with anti-mouse CD45 PerCP-Cy5.5 (clone 30-F11, BioLegend, REF: 103132), anti-mouse CD3 APC (clone 17A2, BioLegend, REF: 100236), anti-mouse CD4 BrilliantViolet 650 (clone GK1.5, BioLegend, REF: 100469), CD8a APC/Cy7 (clone 53-6.7, BioLegend, REF: 100714), anti-mouse CD25 PE (clone PC61, Thermo Fisher, REF: 12-0251-83), anti-mouse CD69 BrilliantViolet 605 (clone H1.2F3, BioLegend, REF: 104530), anti-mouse PD-1 (programmed cell death protein 1) BrilliantViolet 421 (clone 29F.1A12, BioLegend, REF: 109121), anti-mouse/human CD44-AlexaFluor700 (clone IM7, BioLegend, REF: 103026), anti-mouse CD62L-PE/Cy7 (clone MEL-14, Thermo Fisher, REF: 25-0621-82) and anti-mouse/human KLRG1-PE/Dazzle594 (clone 2F1/KLRG1, BioLegend, REF: 138424) for 30 min at 4 °C in the dark. In blood samples, erythrocytes were lysed with BD FACS lysing solution (10 min, 4 °C). After another washing step, Trucount beads were added for cell quantification and the samples were immediately measured using a BD LSR II flow cytometer (Becton Dickinson, USA). In addition, GFP fluorescence was measured.

### FlowJo analysis

Analysis of flow cytometry data was performed using FlowJo (version 10.10.0) (Supplementary Fig. 1). For brain and blood samples, a CD45+ pre-gate was used to exclude debris and make further gating possible. Doublets were excluded by plotting forward scatter (FSC)-W against FSC-H, side scatter (SSC)-W against SSC-H and FSC-H against FSC-A. Then, Zombie+ dead cells were excluded and debris was distinguished from cells by plotting SSC-A against FSC-A. Leukocytes were gated by plotting SSC-A against CD45. In the brain, both CD45intermediate and CD45high events were included in the CD45+ gate. T cells were distinguished via their expression of CD3. CD4+ and CD8+ T cells as well as DNT were distinguished by plotting CD4 against CD8. CD44 and CD62L expression was used to identify naïve (CD44-CD62L+), central memory (CD44+, CD62L+) and effector/effector memory (CD62L-) T cells within overall T cells as well as CD4+, CD8+ and DNT.

KLRG1 and activation markers CD25 and PD-1 were gated manually, while CD69 was gated according to fluorescence minus one (FMO) controls. Due to the limited number of cells, FMO gates from the spleen were used for all organs. The feasibility of this approach was confirmed in comparative experiments during the establishment of staining. A Boolean gate combining all cells expressing at least one of the three activation markers (CD25, CD69, PD-1) was created to evaluate GFP expression on all activated cells. The cutoff between GFP+ and GFP- cells was set according to splenocytes from mice that do not express Nur77^GFP^.

Absolute cell counts were calculated using BD Trucount beads. First, a rough gate was set by plotting SSC-A against FSC-A. From this population beads were distinguished from cells and debris by using the high autofluorescence of the beads in the V670 (CD4) and B530 (GFP) channels.

### Quantification of Aβ-plaques by thioflavine S staining

Thioflavine S is a fluorescent dye that specifically binds to amyloid fibrils and is frequently used to visualize β-Amyloid plaques. The protocol was adapted from Christensen & Pike [52].

After transcardial perfusion, one brain hemisphere was fixed in 4 % paraformaldehyde for 48 h at 4 °C then washed three times with PBS and stored at 4 °C in PBS until sectioning. Brains were sliced at 0.5 mm/s at a depth of 30 μm starting around bregma 0.98 using the Leica semiautomatic vibratome S120. The slices were stored in PBS with 0.03 % sodium azide at 4°C. Six brain sections (every 10th section: bregma -0.46, -0.94, -1.06, -1.45, -1.58, -1.82) were mounted on Superfrost Plus Adhesion Microscope Slides and dried overnight. Then, the slides were dehydrated with 50 % Ethanol (3x 3 minutes), dipped into Milli-Q water and washed in Milli-Q water (3 minutes). Subsequently, slides were incubated in 1 % thioflavine S solution (10 minutes) and washed with 70 % Ethanol (5x 3 minutes), 50 % Ethanol (3x 3 minutes) and Milli-Q water (2x 15 minutes). Slides were dried for 30-60 minutes and then covered with Fluoroshield with DAPI.

For plaque quantification in a section of the entire brain hemisphere, thioflavine S staining was detected in 2x magnification in the GFP channel of a BZ-9000 fluorescence microscope (Keyence) using the BD-2 analyzer software. Exemplary photographs of thioflavine-stained brain sections were taken in 10x magnification.

ImageJ was used for the analysis of Aβ plaque area and number. First, obstacles were removed and the border of the brain slice outlined with the polygon function. Using the “set scale” function, 27.25 pixel were determined as equivalent to 100 µm. The picture was converted to 8-bit. In order to detect only stained plaques, a threshold was set based on wild type brain sections. Then, plaques quantified using the “analyze particle” function. Finally, the median plaque size and density (plaque number/mm^2^) were calculated from all 6 brain sections for each mouse.

### Quantification of Cytokines in the plasma via LEGENDplex

The LEGENDplex Mouse Th Cytokine Panel 12-Plex (BioLegend, REF: 741044, Lot. B426497) was performed according to the manufacturer’s protocol. The standard was performed in duplicates, samples were not diluted and ran in singlets. Instead of vacuuming the beads were centrifuged down at 1100 rpm for 5 minutes and the supernatant was removed via pipette. Samples were measured at a BD LSR II Flow Cytometer in the YG582 and R670 channels. The number of events to detect was set to 5.000 in total. Data analysis was performed in the LEGENDplex software (version 2024-06-15) provided by BioLegend. Data below the detection threshold were set to the corresponding limit of detection of each analyte.

### Sample size and exclusion criteria

In total, we included 69 animals in this study. 13 animals were excluded because they died before their planned endpoint (n=11) or because of flow cytometer malfunction (n=2). This failure rate was covered by the precalculated number of animals needed for the experiments. Sample sizes were calculated with a power of at least 80 % and an alpha level of a maximum of 5 %. The calculation was based on 10,000 Monte Carlo simulations, assuming a drop-out rate of 10 % in the control animals and 30 % (2-6 months) or 50 % (10-18 months) respectively in the Alzheimer's animals.

For the robustness of the data, we excluded data points from flow cytometric analyses when the parent gate did not include sufficient events for further subgating (<50 events in the parent population). Data points of 2 animals were lost due to instrumental failure, additional mice were then tested to replenish data. The accordingly available sample size for each time point and analyses is given in the figure legends. When this led to less than 3 data points per group this group comparison (wt and AD of that time point) was excluded from further analyses. This approach ensures that all data shown and given for interpretation is robust.

### Statistical analysis

Statistical analysis was performed using GraphPad Prism 10.2.3. Kruskal-Wallis test followed by Dunn's multiple comparison post hoc test for selected pairs was used to compare the sum score, Aβ plaque deposition, cytokines, weight and temperature of AD to wt animals for each time point.

For flow cytometric data, outliers in the 1^st^ and 99^th^ percentile were excluded from analysis. Since the first plaques were seen in the 6m AD group, the AD group was compared to wt controls only for the time points 6m, 10-12m and 15-18m. We used Kruskal-Wallis test followed by Dunn's multiple comparison post hoc test for selected pairs. Correlations were calculated using Spearman’s rank-order correlation analysis. Only data from AD mice were correlated. Differences were considered significant at p<0.05.

**Figure 2.**
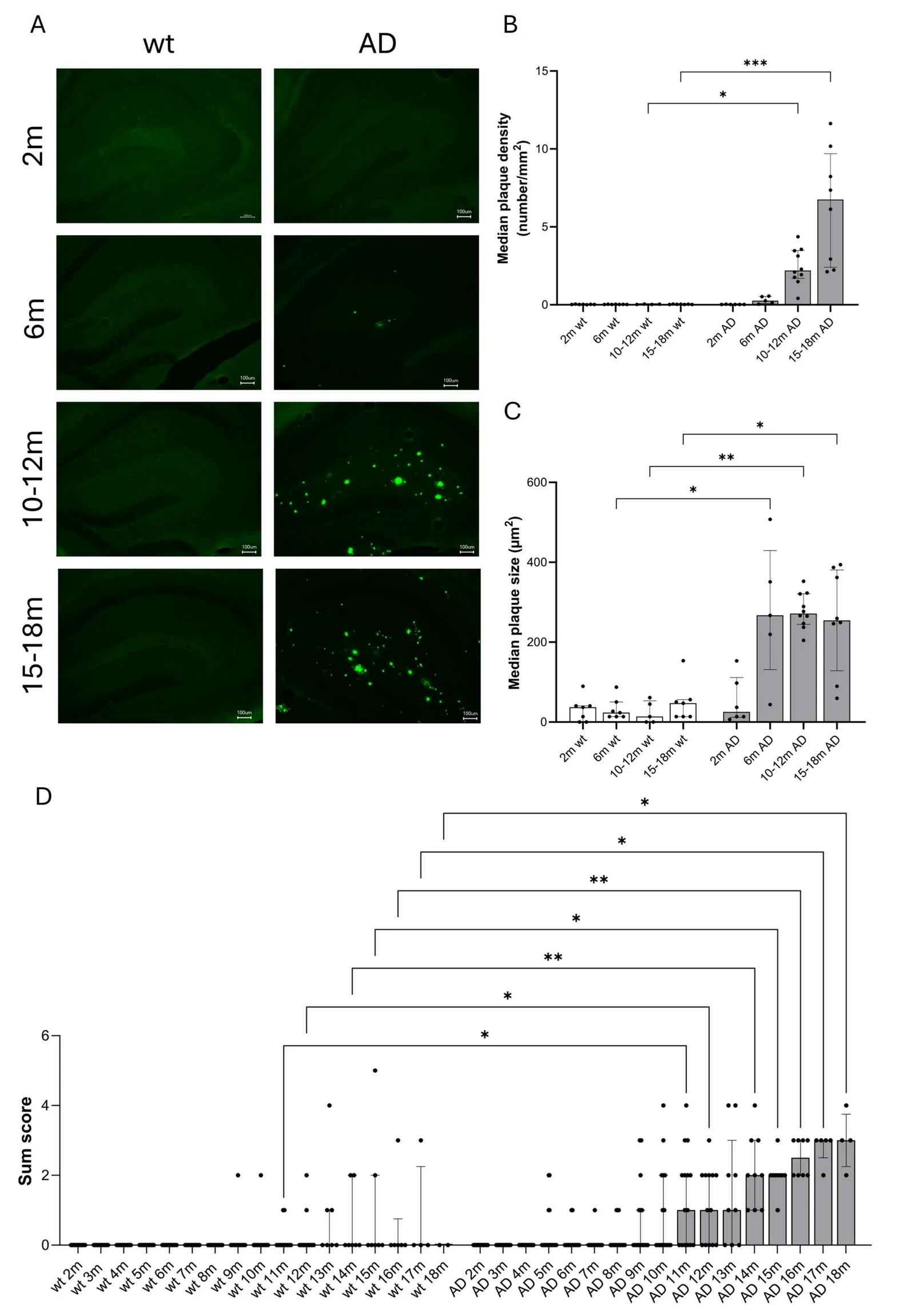
**Development of Alzheimer’s disease (AD)-like amyloid pathology in AD mice**. (**A**) Thioflavine S staining of Aβ plaques in the brains of female wt (Nur77^GFP^) and AD (APP/PS1-Nur77^GFP^) mice in the hippocampal area (bregma -1.45). Thioflavine S staining was detected in 10x magnification in the GFP channel of a BZ-9000 fluorescence microscope (Keyence) using the BD-2 analyzer software. (**B**) Median density of Aβ plaques (number/mm^2^) determined by thioflavine S staining in 6 sections of 1 brain hemisphere (bregma -0.46, -0.94, -1.06, -1.45, -1.58, -1.82) in 2x magnification. *_10-12m_ p=0.0318, ***_15-18m_ p=0.0010. n_2m wt_=7, n_6m wt_=7, n_10-12m wt_=4, n_15-18m wt_=7, n_2m AD_=6, n_6m AD_=5, n_10-12m AD_=10, n_15-18m AD_=8. (**C**) Median size of Aβ plaques determined by thioflavine S staining in 6 sections of 1 brain hemisphere (bregma -0.46, -0.94, -1.06, -1.45, -1.58, -1.82) in 2x magnification. *_6m_ p=0.0335, **_10-12m_ p=0.0018, *_15-18m_ p=0.0303. n_2m wt_=7, n_6m wt_=7, n_10-12m wt_=5, n_15-18m wt_=7, n_2m AD_=6, n_6m AD_=5, n_10-12m AD_=10, n_15-18m AD_=8. (**D**) Sum score consisting of 5 subscores (general condition, fur condition / fur care, activity, posture/movement, induced behavior). *_11m_ p=0.0198; *_12m_ p=0.0422; 13m: not significant; **_14m_ p=0.0074; *_15m_ p=0.0132; **_16m_ p=0.0010; *_17m_ p=0.0427; *_18m_ p=0.0260. n_2m wt_=28, n_3m wt_=21, n_4m wt_=21, n_5m wt_=21, n_6m wt_=20, n_7m wt_=14, n_8m wt_=14, n_9m wt_=14, n_10m wt_=14, n_11m wt_=14, n_12m wt_=14, n_13m wt_=7, n_14m wt_=7, n_15m wt_=7, n_16m wt_=6, n_17m wt_=4, n_18m wt_=2, n_2m AD_=28, n_3m AD_=22, n_4m AD_=22, n_5m AD_=22, n_6m AD_=19, n_7m AD_=17, n_8m AD_=17, n_9m AD_=17, n_10m AD_=17, n_11m AD_=15, n_12m AD_=14, n_13m AD_=9, n_14m AD_=9, n_15m AD_=9, n_16m AD_=8, n_17m AD_=5, n_18m AD_=4. Data are presented as median and IQR. Kruskal-Wallis test, post hoc Dunn’s multiple comparison test for selected pairs (AD vs. wt for each time point), *p < 0.05, **p<0.01, ***p<0.001.

**Figure 3.**
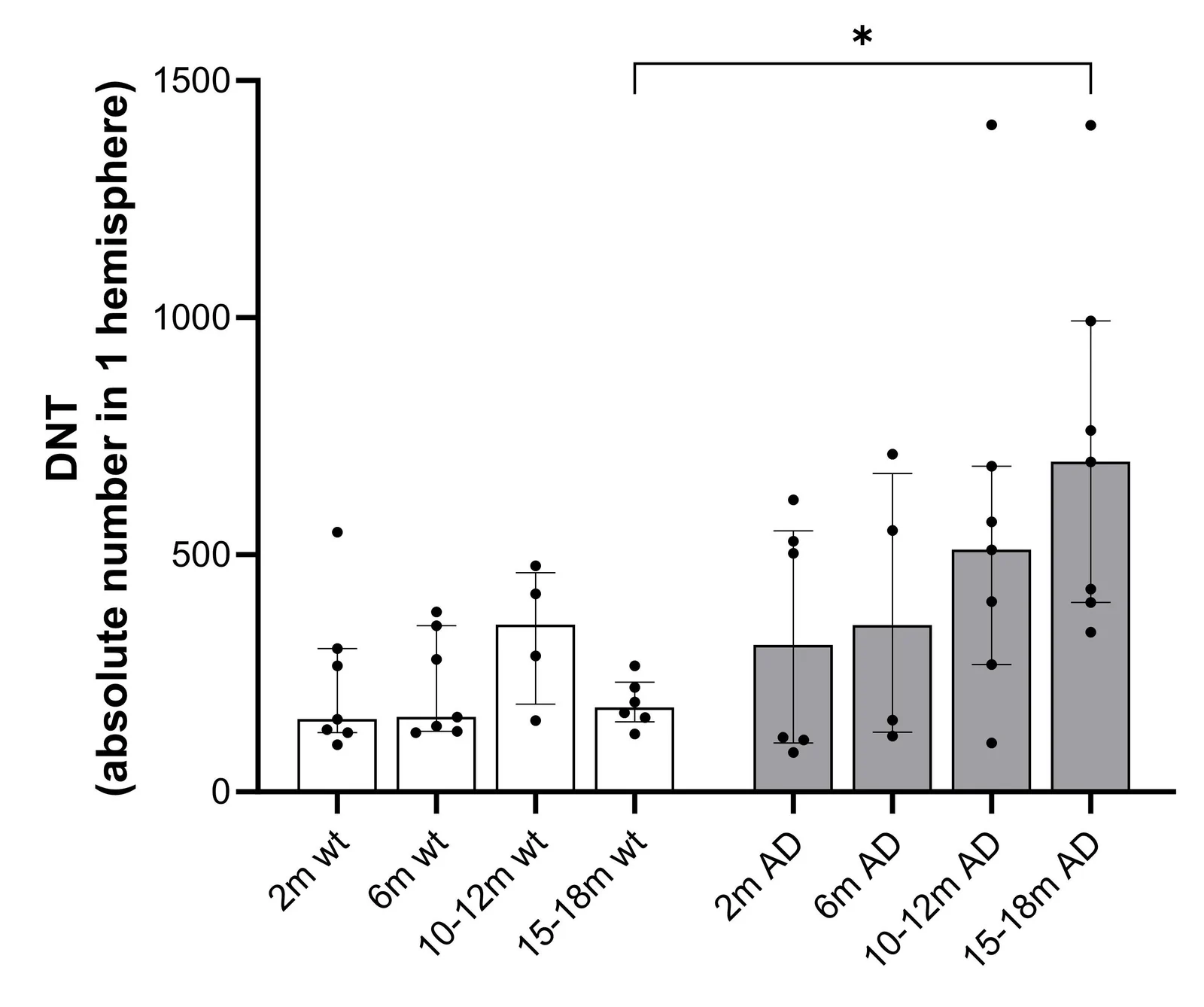
**Increased absolute number of double-negative T cells (DNT; CD4-/CD8-) in the brains (1 hemisphere) of Alzheimer’s disease (AD) mice compared to healthy controls (wt)**. *_15-18m_ p=0.0159. n_2m wt_=7, n_6m wt_=7, n_10-12m wt_=4, n_15-18m wt_=6, n_2m AD_=6, n_6m AD_=4, n_10-12m AD_=7, n_15-18m AD_=7. Data are presented as median and IQR. Kruskal-Wallis test, post hoc Dunn’s multiple comparison test for selected pairs (6m wt vs. 6m AD, 10-12m wt vs. 10-12m AD, 15-18m wt vs. 15-18m AD), *p < 0.05.

## RESULTS

### Data availability statement

All data are available from the authors upon reasonable request.

### AD phenotype

Since no intervention was tested, scores/behavioral testing and plaque formation were only used to verify disease development.

Both average plaque density and size of amyloid β plaques were significantly increased in AD mice compared to wildtype at the age of 6 (only plaque size: p=0.0335) 10-12 (p_density_=0.0318, p_size_=0.0018) and 15-18 months (p_density_=0.0010, p_size_=0.0303) (Fig. 2A-C) and the plaque density correlated with the absolute number of T cells in the brain (Spearman’s r=0.5991, p=0.0020; data not shown).

Significant differences in the sum score consisting of 5 subscores (general condition, fur condition/fur care, activity, posture/movement, induced behavior, supplementary S1) were only apparent between the age of 11 and 18 months (Fig. 2D). There was no significant difference in body weight or temperature between AD and wt animals (data not shown).

### Higher DNT counts in the AD brain

At 15-18 months, DNT numbers in the brain were significantly increased in AD mice compared to age-matched controls (Fig. 3). There was no significant difference in number or percentage of CD4+ or CD8+ T cells in the brain or in other investigated organs (data not shown).

**Figure 4.**
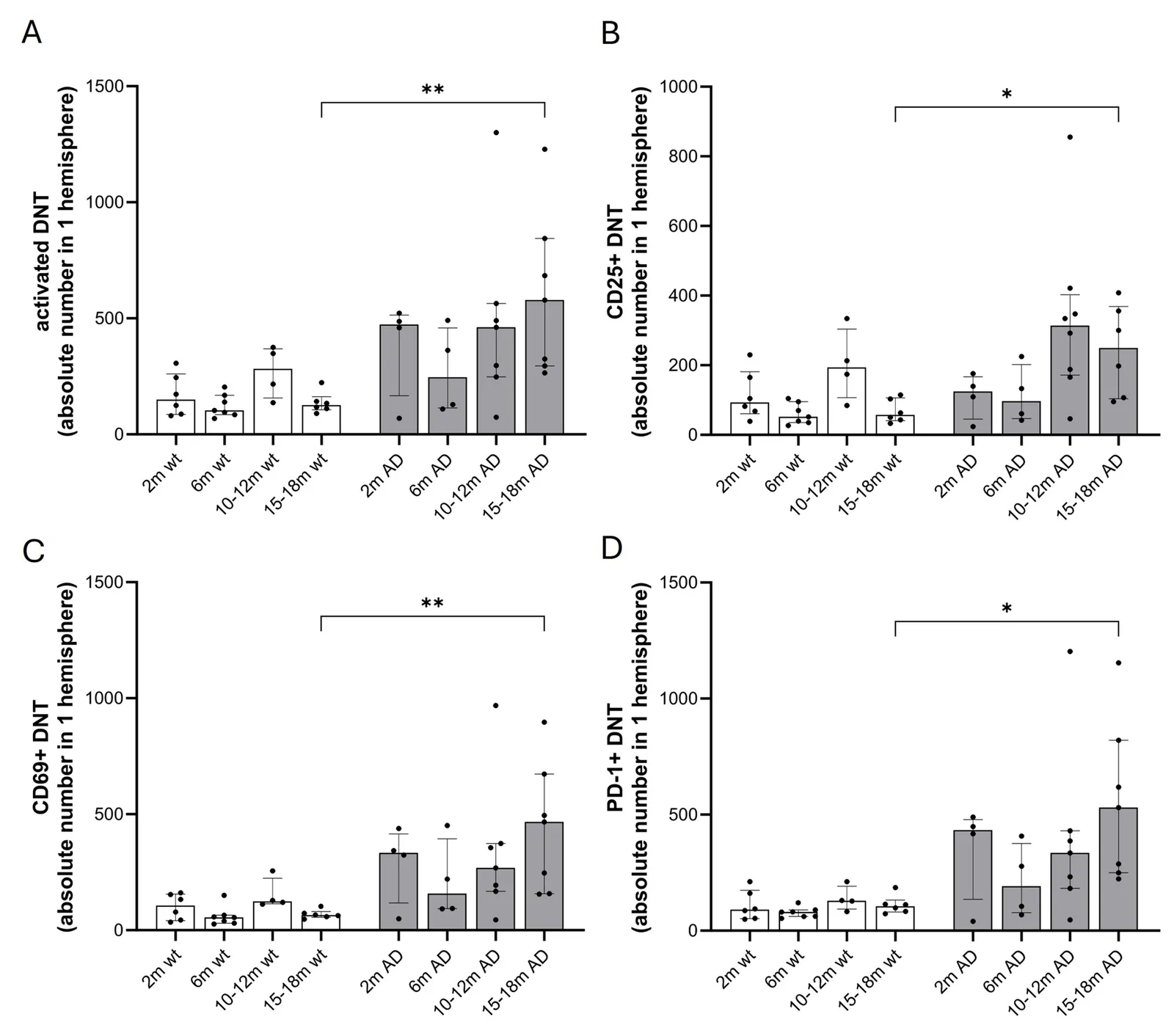
**Increased numbers of activated double-negative T cells (DNT; CD4-/CD8-) in the brains (1 hemisphere) of Alzheimer’s disease (AD) mice compared to healthy controls (wt)**. (**A**) Absolute number of overall activated DNT (expressing CD25 and/or CD69 and/or PD-1). **_15-18m_ p=0.0085. n_2m wt_=6, n_6m wt_=7, n_10-12m wt_=4, n_15-18m wt_=6, n_2m AD_=4, n_6m AD_=4, n_10-12m AD_=7, n_15-18m AD_=7. (**B**) Absolute number of CD25+ DNT. *_15-18m_ p= 0.0267, n_2m wt_=6, n_6m wt_=7, n_10-12m wt_=4, n_15-18m wt_=6, n_2m AD_=4, n_6m AD_=4, n_10-12m AD_=8, n_15-18m AD_=6. (**C**) Absolute number of CD69+ DNT. **_15-18m_ p=0.0040, n_2m wt_=6, n_6m wt_=7, n_10-12m wt_=4, n_15-18m wt_=6, n_2m AD_=4, n_6m AD_=4, n_10-12m AD_=7, n_15-18m AD_=7. (**D**) Absolute number of PD-1+ DNT. *_15-18m_ p=0.0168, n_2m wt_=6, n_6m wt_=7, n_10-12m wt_=4, n_15-18m wt_=6, n_2m AD_=4, n_6m AD_=4, n_10-12m AD_=7, n_15-18m AD_=7. Data are presented as median and IQR. Kruskal-Wallis test, post hoc Dunn’s multiple comparison test for selected pairs (6m wt vs. 6m AD, 10-12m wt vs. 10-12m AD, 15-18m wt vs. 15-18m AD), *p < 0.05, **p < 0.01.

**Figure 5.**
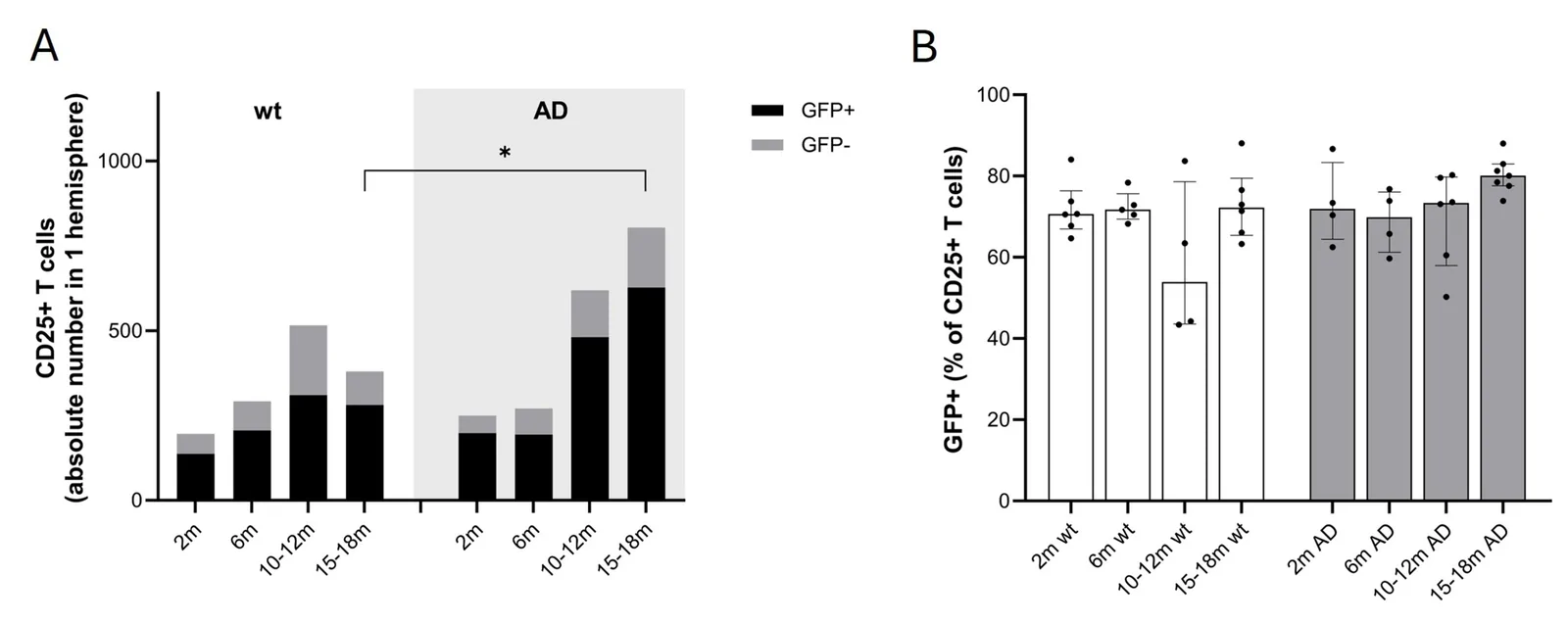
**GFP expression is increased on CD25+ T cells within the brains (1 hemisphere) of Alzheimer’s disease (AD) mice compared to healthy controls (wt)**. (**A**) Absolute number of GFP+ (dark gray) and GFP- (light gray) CD25+ T cells. This figure depicts the contribution of antigen-specific (dark gray) and -unspecific (light gray) activation. Both alternatives have therefore been stacked. *_15-18m_ p=0.0317. n_2m wt_=6, n_6m wt_=4, n_10-12m wt_=5, n_15-18m wt_=4, n_2m AD_=4, n_6m AD_=7, n_10-12m AD_=6, n_15-18m AD_=6. (**B**) Proportion of GFP+ cells within CD25+ T cells. n_2m wt_=6, n_6m wt_=5, n_10-12m wt_=4, n_15-18m wt_=6, n_2m AD_=4, n_6m AD_=4, n_10-12m AD_=6, n_15-18m AD_=7. Data information: Data are shown as medians. Kruskal-Wallis test, post hoc Dunn’s multiple comparison test for selected pairs (6m wt vs. 6m AD, 10-12m wt vs. 10-12m AD, 15-18m wt vs. 15-18m AD, *p < 0.05).

### Increased numbers of activated double-negative T cells (DNT) in the brain of AD mice

Although the percentage of activated DNT (characterized by the expression of CD25 and/or CD69 and/or PD-1) within overall DNTs in the brains of AD and wt mice was comparable, the absolute number of activated DNT was significantly higher in AD mice at 15-18 months. This increase was observed for overall activated DNT (Fig. 4A), as well as for CD25+, CD69+, and PD-1+ DNT subsets (Fig. 4B-D). No statistically significant differences were found in the expression of activation markers in brain CD4+ or CD8+ T cell populations or in other investigated organs (data not shown).

**Figure 6.**
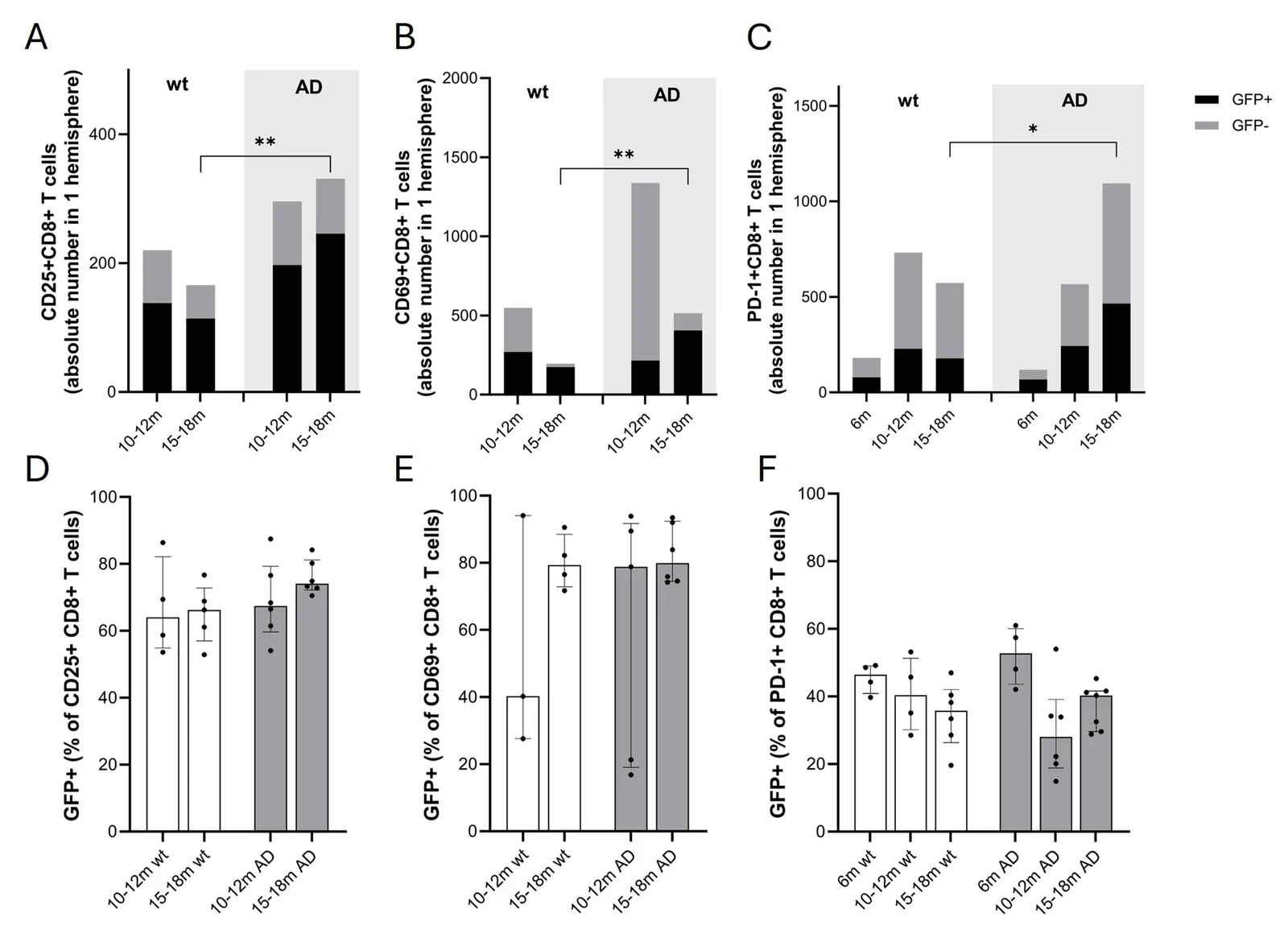
**GFP expression is increased on CD8+ T cells within the brains (1 hemisphere) of Alzheimer’s disease (AD) mice compared to healthy controls (wt)**. The figures A-C depict the contribution of antigen-specific (dark gray) and -unspecific (light gray) activation. Both alternatives have therefore been stacked. If fewer than three data points remained in a group after exclusion—due to unreliable gating caused by low event counts or indistinguishable populations—the corresponding time point was omitted from the analysis. (**A**) Absolute number of GFP+ (dark gray) and GFP- (light gray) CD25+ CD8+ T cells. **_15-18m_ p=0.0019. n_10-12m wt_=4, n_15-18m wt_=7, n_10-12m AD_=4, n_15-18m AD_=7. (**B**) Absolute number of GFP+ (dark gray) and GFP- (light gray) CD69+ CD8+ T cells. **_15-18m_ p=0.0071. n_10-12m wt_=3, n_15-18m wt_=3, n_10-12m AD_=3, n_15-18m AD_=6. (**C**) Absolute number of GFP+ (dark gray) and GFP- (light gray) PD-1+ CD8+ T cells. *_15-18m_ p=0.0162. n_6m wt_=4, n_10-12m wt_=4, n_15-18m wt_=4, n_6m AD_=6, n_10-12m AD_=6, n_15-18m AD_=6. (**D**) Proportion of GFP+ cells within CD25+ CD8+ T cells. n_10-12m wt_=4, n_15-18m wt_=5, n_10-12m AD_=6, n_15-18m AD_=6. (**E**) Proportion of GFP+ cells within CD69+ CD8+ T cells. n_10-12m wt_=3, n_15-18m wt_=4, n_10-12m AD_=5, n_15-18m AD_=6. F Proportion of GFP+ cells within PD-1+ CD8+ T cells. n_6m wt_=4, n_10-12m wt_=4, n_15-18m wt_=6, n_6m AD_=4, n_10-12m AD_=6, n_15-18m AD_=7. Data information: Data are shown as medians. Kruskal-Wallis test, post hoc Dunn’s multiple comparison test for selected pairs (6m wt vs. 6m AD, 10-12m wt vs. 10-12m AD, 15-18m wt vs. 15-18m AD, *p < 0.05, **p < 0.01).

### Increased GFP expression in cerebral T cells in AD

**Figure 7.**
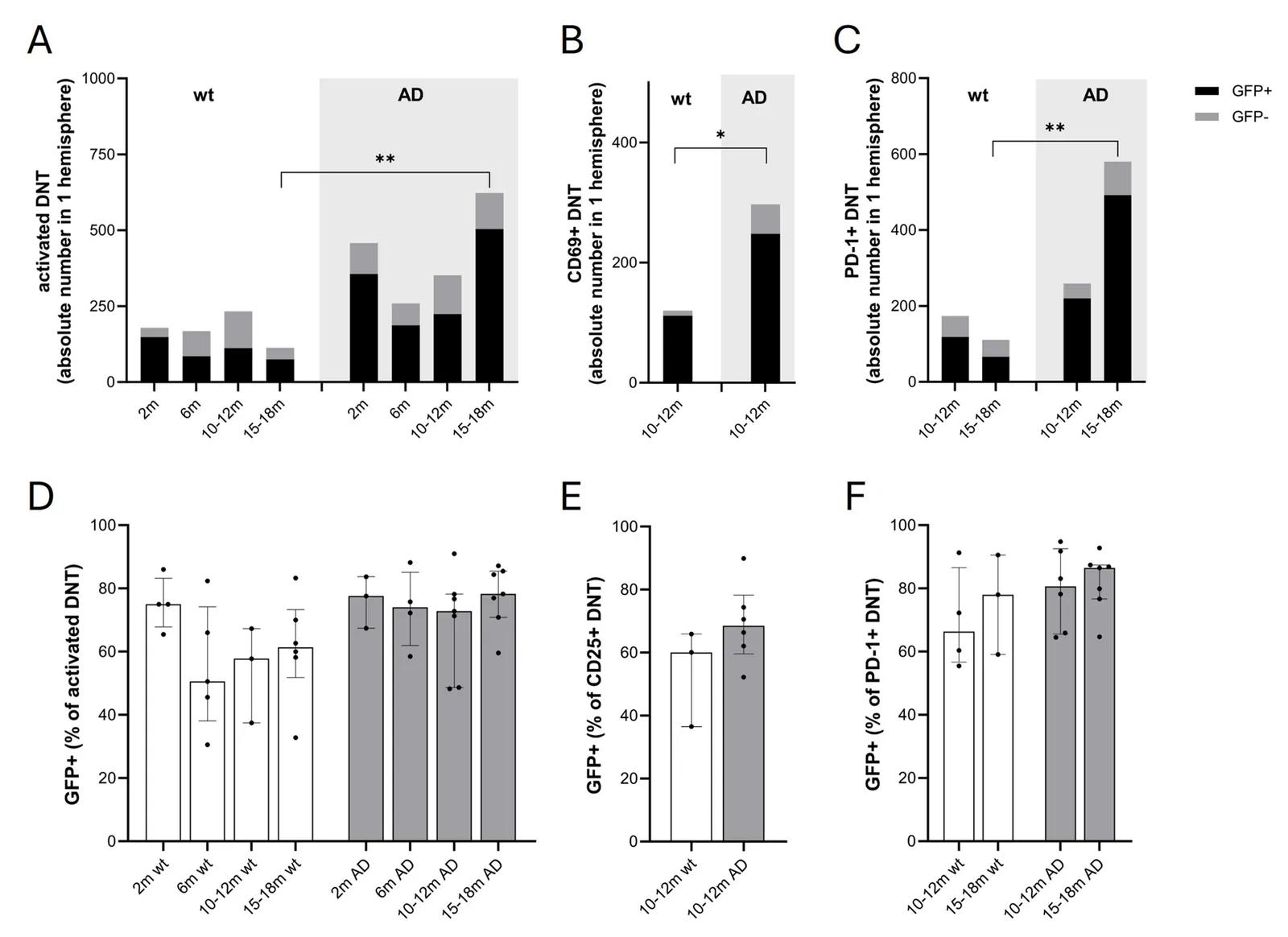
**GFP expression is increased on double-negative T cells (DNT; CD4-/CD8-) within the brains (1 hemisphere) of Alzheimer’s disease (AD) mice compared to healthy controls (wt)**. The figures A-C depict the contribution of antigen-specific (dark gray) and -unspecific (light gray) activation. Both alternatives have therefore been stacked. If fewer than three data points remained in a group after exclusion—due to unreliable gating caused by low event counts or indistinguishable populations—the corresponding time point was omitted from the analysis. (**A**) Absolute number of GFP+ (dark gray) and GFP- (light gray) activated DNT (expressing CD25 and/or CD69 and/or PD-1). **_15-18m_ p=0.0013. n_2m wt_=5, n_6m wt_=3, n_10-12m wt_=3, n_15-18m wt_=4, n_2m AD_=4, n_6m AD_=5, n_10-12m AD_=6, n_15-18m AD_=7. (**B**) Absolute number of GFP+ (dark gray) and GFP- (light gray) CD69+ DNT. *_10-12m_ p= 0.0476. n_10-12m wt_=3, n_10-12m AD_=6. (**C**) Absolute number of GFP+ (dark gray) and GFP- (light gray) PD-1+ DNT. **_15-18m_ p=0.0025. n_10-12m wt_=3, n_15-18m wt_=5, n_10-12m AD_=4, n_15-18m AD_=7. (**D**) Proportion of GFP+ cells within activated DNT. n_2m wt_=4, n_6m wt_=5, n_10-12m wt_=3, n_15-18m wt_=6, n_2m AD_=3, n_6m AD_=4, n_10-12m AD_=7, n_15-18m AD_=7. (**E**) Proportion of GFP+ cells within CD69+ DNT. n_10-12m wt_=3, n_10-12m AD_=6. (**F**) Proportion of GFP+ cells within PD-1+ DNT. n_10-12m wt_=4, n_15-18m wt_=3, n_10-12m AD_=6, n_15-18m AD_=7. Data information: Data are shown as medians. Kruskal-Wallis test, post hoc Dunn’s multiple comparison test for selected pairs (6m wt vs. 6m AD, 10-12m wt vs. 10-12m AD, 15-18m wt vs. 15-18m AD) or Mann-Whitney-U test. *p < 0.05, **p < 0.01.

The absolute number of GFP+ CD25+ T cells (Fig. 5) as well as GFP+ CD25+ CD8+ T cells (Fig. 6A) was increased in the brain of AD mice compared to healthy mice. During the 15-18 months, AD mice also exhibited a significantly higher number of GFP+ CD69+ (Fig. 6B) and GFP+ PD-1+ CD8+ T cells (Fig. 6C). Additionally, the absolute number of GFP-expressing activated DNT (Fig. 7A) and GFP+ PD-1+ DNT (Fig. 7C) were markedly elevated in AD animals at the age of 15-18 months. At 10-12 months, the absolute number of GFP+CD69+ DNT (Fig. 7B) was significantly increased in AD mice. Peripheral analysis of cervical lymph nodes (Supplementary Fig. 2), spleen, and blood (data not shown) revealed negligible changes, indicating that the primary effects are localized to the brain.

### More highly differentiated DNT

**Figure 8.**
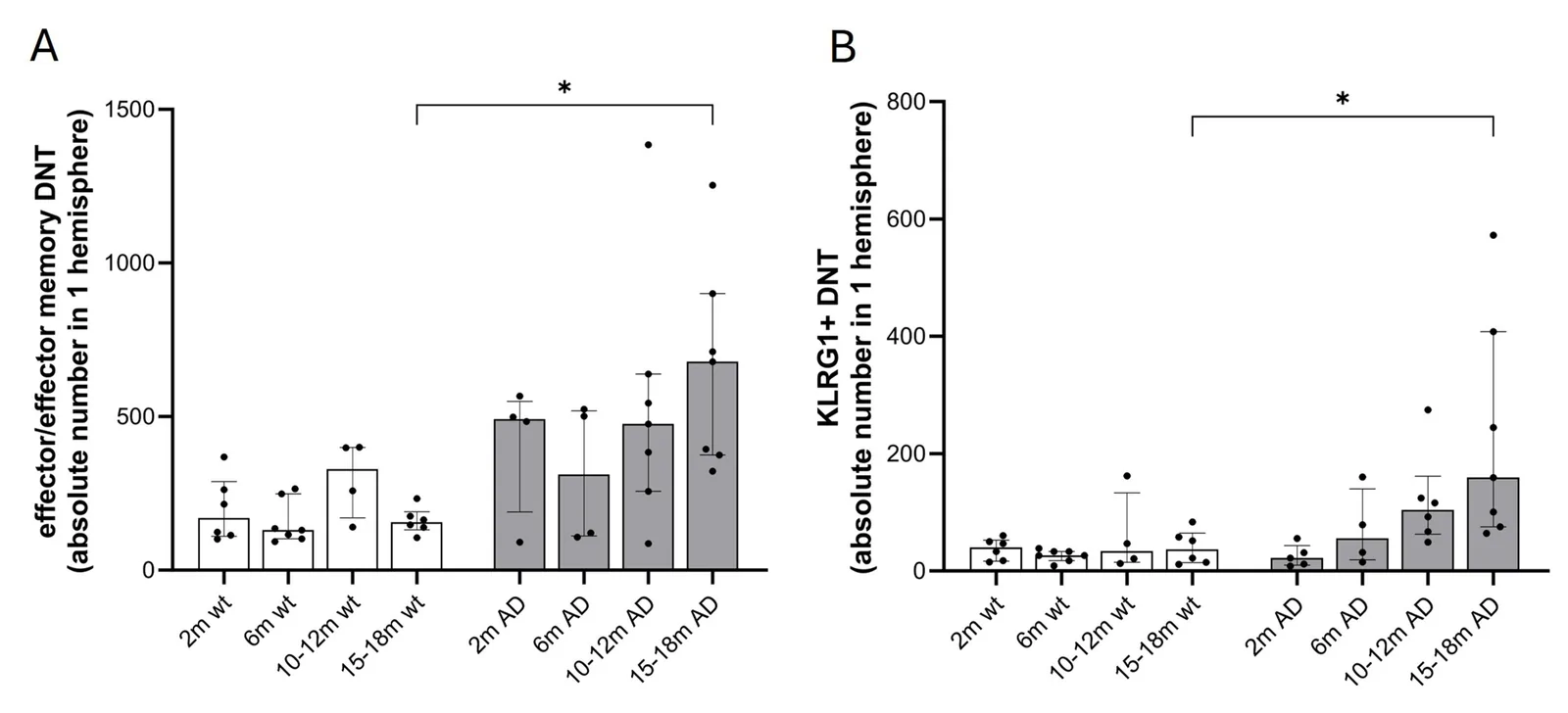
**Increase in highly differentiated double-negative T cells (DNT; CD4-/CD8-) within the brains (1 hemisphere) of Alzheimer’s disease (AD) mice compared to healthy controls (wt)**. (**A**) Absolute number of overall effector / effector memory DNT. *_15-18m_ p=0.0115. n_2m wt_=6, n_6m wt_=7, n_10-12m wt_=4, n_15-18m wt_=6, n_2m AD_=4, n_6m AD_=4, n_10-12m AD_=7, n_15-18m AD_=7. (**B**) Absolute number of KLRG1+ DNT. *_15-18m_ p=0.0190. n_2m wt_=6, n_6m wt_=7, n_10-12m wt_=4, n_15-18m wt_=6, n_2m AD_=5, n_6m AD_=4, n_10-12m AD_=6, n_15-18m AD_=7. Data information: Data are presented as median and IQR. Kruskal-Wallis test, post hoc Dunn’s multiple comparison test for selected pairs (6m wt vs. 6m AD, 10-12m wt vs. 10-12m AD, 15-18m wt vs. 15-18m AD, *p < 0.05).

In the brain of 15-18 months old AD mice, the absolute number of effector/effector memory DNT and KLRG1+ DNT was significantly increased compared to wt animals (Fig. 8).

### Cytokine profile in the plasma

**Figure 9.**
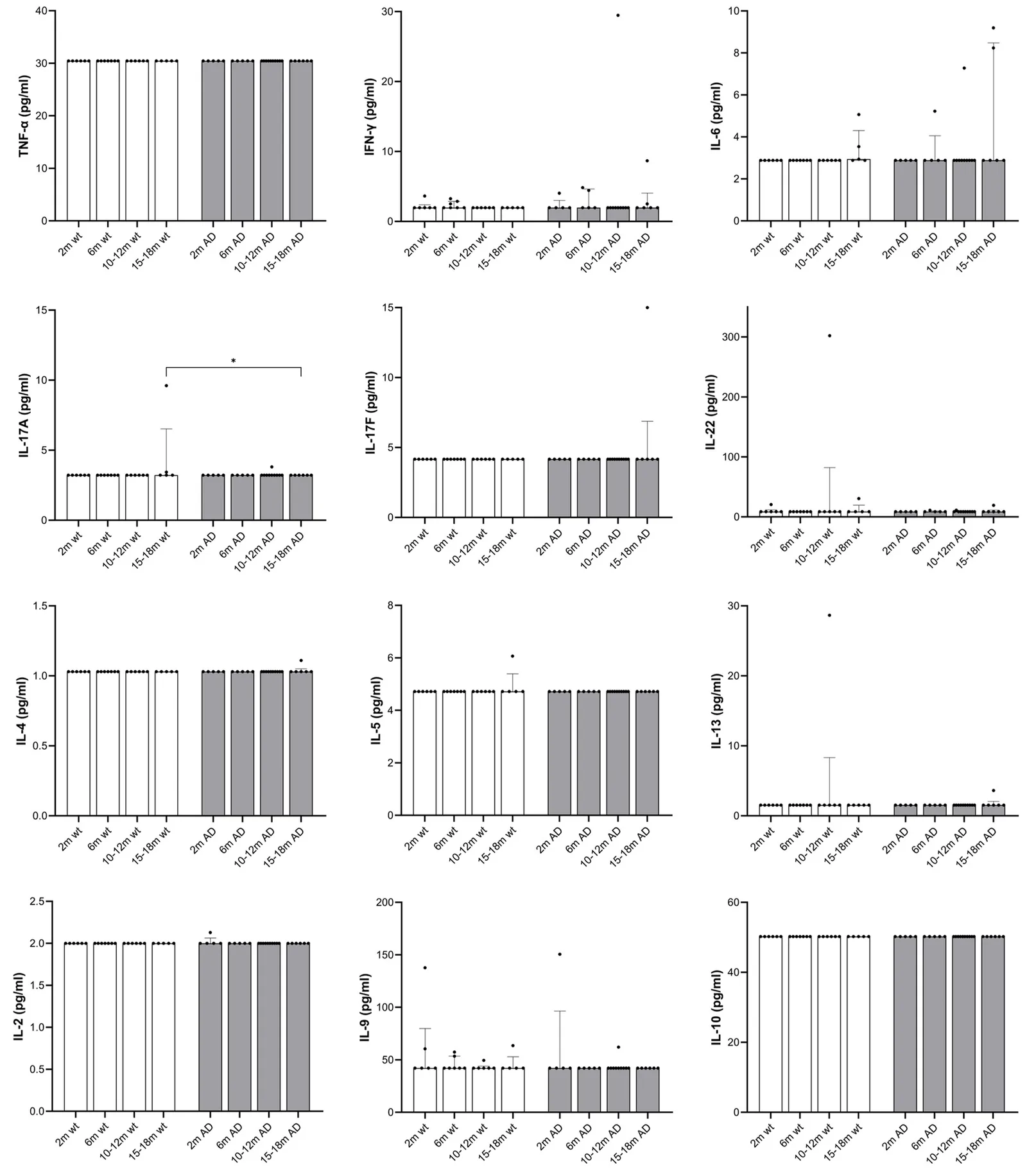
**Cytokine profile in the plasma of Alzheimer’s disease (AD) mice compared to healthy controls (wt)**. n_2m wt_=6, n_6m wt_=7, n_10-12m wt_=4, n_15-18m wt_=6, n_2m AD_=5, n_6m AD_=4, n_10-12m AD_=8, n_15-18m AD_=7. Data information: Data below the detection threshold were set to the corresponding limit of detection of each analyte. Data are presented as median and IQR. Kruskal-Wallis test, post hoc Dunn’s multiple comparison test for selected pairs (6m wt vs. 6m AD, 10-12m wt vs. 10-12m AD, 15-18m wt vs. 15-18m AD, *p < 0.05).

Cytokine levels in the plasma were below the detection limit for most animals across all ages in both WT and AD groups. As a result, significant differences in cytokine expression were generally undetectable, with the exception of IL-17A, which was found to be significantly elevated in 15-18-month-old wild-type mice compared to AD mice (Fig. 9). However, it is important to note that this difference was driven by a single data point.

## DISCUSSION

Inflammation is a key driver of AD pathology, contributing to the accumulation of amyloid-beta plaques and tau tangles, activating glial cells, and driving neurodegeneration. We observed elevated numbers of activated DNT in the AD brain, suggesting an increased proinflammatory environment. By combining the APP/PS1 AD mouse model with the Nur77^GFP^ model, we were able to identify and track antigen-specific T cell activation during disease progression within the different T cell subsets. The exclusive increase of the absolute amount of antigen-specifically activated cells highlights their potential as therapeutic targets. Interestingly, the primary immunological changes were confined to the brain while peripheral effects in lymphoid tissues and blood (T cell activation and cytokine expression) were minimal, emphasizing the localized nature of immune activation in AD.

In contrast to other studies [16-19] we did not observe an overall increase in CD8+ T cells in AD mice compared to controls, but there was an increase in absolute numbers of antigen-specifically activated CD25+, CD69+ and PD-1+ CD8+ T cells in AD brains (Fig. 6A-C). Previous research has shown that activated T cells are significantly increased in the brain, cerebrospinal fluid and peripheral blood during AD [13-15]. CD8+ T cells have been shown to potentiate amyloid deposition and the activation of CD8+ T cells has been linked to microstructural damage in parahippocampal areas as well as neurological deficits [12-16, 44, 53]. Further research has to evaluate if the increase of antigen-specifically activated CD8+ T cells found in our study has beneficial or detrimental consequences.

Previous studies have also observed differences in DNT primarily in the peripheral blood and spleen of AD patients and in the APP/PS1 and 3xTg AD mouse models [36, 38-40], where DNT were significantly elevated and linked to cognitive deficits. However, in our APP/PS1-Nur77^GFP^ mice, we did not replicate an increase in peripheral DNT. Instead, we observed a significant increase in the absolute number of DNT, particularly activated (expressing CD25 and/or CD69 and/or PD-1) DNT, in the brains of AD animals compared to healthy controls (Fig. 3, 4).

In addition to T cells escaping from negative selection in the thymus [54] DNT may develop in the periphery from (chronically) activated CD4+ or CD8+ T cells that down-regulate their CD4 or CD8 molecules [55-60]. There is evidence, that an especially proinflammatory subset of DNTs is derived from self-reactive CD8+ T cells [61, 62]. In line with that we found high numbers of antigen-specifically activated PD-1+ DNT in the brains of AD mice (Fig. 7C), that possibly increase inflammation and autoimmune activities.

Circulating DNT have generally been regarded as detrimental in AD pathology as they enhance the proinflammatory milieu by secreting TNFα, activating the NLRP3 inflammasome and promoting microglial M1 polarization in vitro [36, 37]. Also, DNT have been shown to exacerbate brain tissue damage in ischemic stroke [63]. These findings suggest that an accumulation of DNT and especially activated DNT in the brain contributes to neuronal loss and aggravates functional deficits in AD pathology. Further research on infiltrated DNT in AD is urgently needed to determine their function and role in disease pathology and their mode of activation to eventually identify specific treatment targets. First studies in that area have shown that short chain fatty acids (SCFA), that are also produced by gut microbiota, increase intestinal DNT, induce the NLRP3 inflammasome and worsen cognitive function in AD mice while silencing NLRP3 reversed DNT-related deficits [36, 37, 64].

It has been shown that in AD, there is an increase in highly differentiated T cell phenotypes like memory and effector memory cells while naïve T cells are decreased compared to controls [12, 13, 15, 16, 65, 66]. Consistent with previous findings, effector/effector memory DNT cells were elevated in the brains of APP/PS1-Nur77 mice (Fig. 8A). Senescent T cells are antigen-experienced cells with diminished responsiveness to new antigens, which secrete proinflammatory cytokines and contribute to chronic low-grade inflammation, a phenomenon known as inflammaging [67]. Our data show an increase in KLRG1+ DNT cells (Fig. 8B), a marker of senescence and terminal differentiation that has been reported to be upregulated on CD4+ T cells in the blood of patients with AD and increased on brain CD8+ T cells in AD models [13, 44].

We observed elevated numbers of antigen-specifically activated PD-1+ CD8+ T cells and PD-1+ DNT in the brain (Fig. 6C, 7C). PD-1/PD-L1 signaling suppresses T cell activation, proliferation and cytokine production as well as memory T cell responses [68-70]. PD-1+ cells have been shown to produce the majority of proinflammatory cytokines within DNT [62]. In the AD brain, PD-1+ CD8+ T cells are increased, inhibit proinflammatory activities of microglia and limit Aβ deposition and cognitive impairment [18]. On the other hand, blockade of the PD-1/PD-L1 pathway also resulted in an increased clearance of amyloid plaques and improved cognitive function in different AD mouse models [71, 72].

PD-1 is viewed as a marker of T cell exhaustion. Chronic inflammation and overstimulation leads to differentiated cells being functionally defective with impaired cytokine production and an inability to adequately respond to antigens [73]. In individuals with β-amyloid depositions, patients with mild cognitive impairment had significantly more exhausted T cells than cognitively normal patients [12]. Given that mostly antigen-specifically activated PD-1+CD8+ T cells were increased, and that differences in T cell activation were seen almost exclusively in the brain, an overstimulation by brain-specific antigens is conceivable. Recently, PD-1 has been suggested as a marker of brain-resident T cells [18, 74, 75]. Tissue-resident memory T cells are mainly found at sites of current or previous inflammation in peripheral organs including the brain, where they can be reactivated and rapidly respond to their cognate antigen [76, 77]. Although they express high levels of inhibitory receptors like PD-1, they are able to produce proinflammatory cytokines and activate myeloid cells upon sufficient stimulation [74, 78]. Since the immune changes we observed are confined to the brain, it would be informative to quantify local cytokine levels in addition to cytokine levels in the plasma (Fig. 9). In addition to investigating the role of pro- and anti-inflammatory brain-resident T cells, determining whether they are truly exhausted or still respond to antigenic stimulation will be imperative to developing the most effective treatment approach.

Innate and adaptive autoimmune reactions have been shown to be present in AD and a recent hypothesis on the underlying pathomechanism even describes AD as an “innate autoimmune disease” [79]. AD goes along with altered levels of autoantibodies and increased T cell reactivity against a variety of antigens, but their functional role is still unclear [16, 41, 80-83]. Previous research has mostly detected antigen-specific cells by looking for reactivity against a predetermined antigen, possibly overlooking so far unknown antigens. Here, we utilized the APP/PS1- Nur77^GFP^ mouse model, which allows detection and quantification of antigen-specifically activated lymphocytes independently of the nature of the antigen. At the age of 15-18 months, we found significantly higher numbers of GFP+ and thus antigen-specifically activated CD25+ T cells (Fig. 5) and CD25+ CD8+ T cells (Fig. 6A). The same was true for CD69+ CD8+, PD-1+ CD8+ (Fig. 6B, C) and PD-1+ DNT as well as overall GFP+ activated DNT (Fig. 7A, C). Our findings support the previously reported involvement of autoimmunity in AD and closer characterization of the phenotype and antigen-specificity of the identified T cell subsets will greatly advance our understanding of the AD pathomechanism.

## Limitations:

One limitation of this study is the nature of the APP/PS1-Nur77^GFP^ mouse model, that only induces amyloid pathology while tau depositions do not develop. This may not fully replicate the complexity of AD pathology in humans but does resemble important aspects of the AD pathology. Validation in human tissues is necessary to confirm these findings. All immune cells have a constant turnover and experimental data represent mere snapshots of an everchanging process. Although T cells that have been activated antigen-specifically more than 3 days before measurement could not be detected due to the transient expression of GFP after TCR engagement, our data still deliver valuable insights into the given immune repertoire. We used female mice only to reduce animal numbers, since the onset and course of the disease varies between sex in the APP/PS1 model. Our findings will need to be validated in male mice. The exclusion of certain data points due to low event counts or indistinguishable populations may also limit the comprehensiveness of our analysis. Lastly, while the study provides important insights into antigen-specific T cell activation, further functional tests and mechanistic experiments are needed to assess the impact of antigen-specifically activated T cells on AD progression.

## Conclusions:

Our data show the increased presence of antigen-specifically activated T cells in the brains of mice developing AD-like amyloid pathology, highlighting their potential role in AD. We gained insights into the entire antigen-specifically activated T cell repertoire, unrestricted by previously identified AD-relevant antigens. At the age of 15- 18 months, we found significantly higher numbers of antigen-specifically activated T cells - especially antigen-specifically activated CD8+ T cells and DNT - in the brains of AD mice. Given their increased presence in the brain, these antigen-specifically activated T cell subsets could represent a promising target for immunomodulatory treatments in AD. The fact that a significant increase in the absolute amount of antigen-specifically activated T cells is found at later stages of the disease argues that therapeutic strategies might have to be adapted during the disease course.

To date, immunomodulatory treatment approaches have not yielded significant improvements in AD outcomes. However, strategies targeting antigen-specifically or antigen-unspecifically activated cells depending on the disease progression may offer promising new therapeutic avenues. Further research is essential to fully characterize these activated T cells, identify their specific antigens, and better understand their role in the complex pathogenesis of AD.

## Supplementary Materials

The Supplementary data can be found online at: www.aginganddisease.org/EN/10.14336/AD.2025.0452.


