# Supplementary material for: Antigen-Specifically Activated CD8+ and Double-Negative T Cells Accumulate in the Brain of Alzheimer’s Disease Mice
Source: Aging Dis. 2025 Jun 27;17(4):2181–97. doi: 10.14336/AD.2025.0452 (PMC13256553; doi:10.14336/AD.2025.0452)
Supplement: Supplementary file 1 [file AD-17-4-2181-s.pdf]

SUPPLEMENTARY DATA

Antigen-Specifically Activated CD8+ and Double-Negative T Cells Accumulate in the Brain of Alzheimer's Disease Mice

Juliane Gellrich, Johanna Ruhnau, Lea Köslich, Stefan Gross, Agnes Flöel, Juliane Schulze, Antje Vogelgesang

© 2025. Gellrich J. et al. Published online at http://www.aginganddisease.org/EN/10.14336/AD.2025.0452

SUPPLEMENTARY DATA

Supplementary Table 1. Sum score

General condition	Healthy appearance	0
	Flanks slightly sunken in	1
	Flanks clearly sunken in	2
	Cachexia, visible spine, moribund	3/h.E.
Fur condition	normal, well-groomed	0
	Slightly unkempt fur	1
	Slight piloerection	2
	Obvious piloerection, dirty fur, dehydration	3/h.E.
Activity	normal, lively, curious	0
	Less lively/curious or hyperactive	1
	Tense/nervous during handling, reduced mobility, reduced alertness	2
	Clearly disturbed during handling, absolute immobility, separation	3/h.E.
Posture / movement	Normal posture and gait, alert eyes, ears, erect ears	0
	Slightly altered gait with normal motorfunction, slightly squinted eyes, ears slightly laid back	1
	Slight deficits in motorfunction	2
	Ataxia, eyes squinted/almost closed, ears laid back, tremor or repeated convulsions, hunched posture	3/h.E.
IV- induced behavior (flight & grooming behavior in response to a water droplet on the nose)	Moves away quickly, instant grooming behavior	0
	Moves away slowly, slightly delayed grooming behavior	1
	Delayed movement or exaggerated reaction, delayed/reduced grooming behavior	2
	No movement or grossly exaggerated reaction, no grooming behavior	3/h.E.

S2 Tissue processing

S2.1 Spleen tissue and CLN processing

Spleens and CLN were mechanically homogenized on a 70-µm cell strainer (Greiner bio-one) with the plunger of a 2 ml syringe, flushing with HBSS (w/o Ca/Mg). After centrifugation (300xg, 5 min), CLN samples were resuspended in HBSS (w/o Ca/Mg) for flow cytometry staining and only spleen cell pellets were resuspended in 2 ml of HBSS (w/o Ca/Mg) and incubated with 5 ml of ACK (Ammonium-Chloride-Potassium) lysis buffer (Gibco) for 10 min at 4°C for erythrocyte lysis. Cells were washed and resuspended in HBSS (w/o Ca/Mg) for flow cytometry staining.

S2.2 Blood processing

Blood samples were immediately transferred to 1.3 ml blood collection microtubes (Sarstedt, Germany) for EDTA anticoagulation. Blood parameters were measured using a VetScan HM5 (Abaxis, USA). The following parameters were detected: leukocytes (absolute), lymphocytes (absolute and relative), monocytes (absolute and relative), neutrophils (absolute and relative), red blood count, hemoglobin [g/dl], hematocrit, mean cell volume, mean cell hemoglobin, mean cell hemoglobin concentration, red cell distribution width, platelets, platelet hematocrit, mean platelet volume and platelet distribution width.

100 µL of EDTA-anticoagulated blood were transferred to a FACS tube, and flow cytometry staining was performed. After extracellular staining, erythrocytes were lysed for 10 min at 4°C in BD FACS lysis solution. Plasma was prepared from the remaining blood by centrifugation (1500g, 5 min, 4°C) and frozen at -80°C for further analyses.

© 2025. Gellrich J. et al. Published online at http://www.aginganddisease.org/EN/10.14336/AD.2025.0452

SUPPLEMENTARY DATA

S3 Gating strategy for flow cytometric data

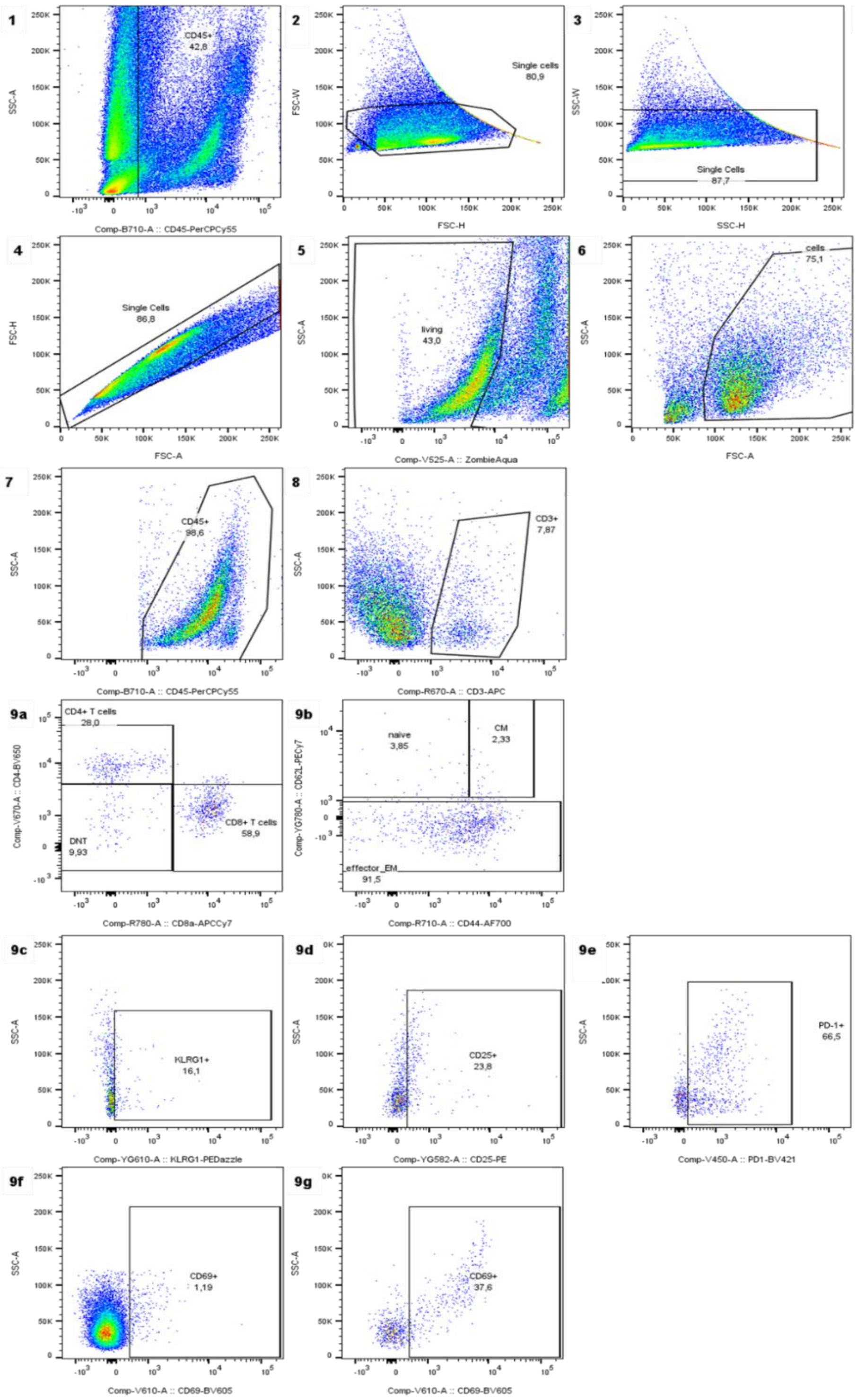

Supplementary Figure 1. Gating strategy for flow cytometric data.
Data were analyzed using FlowJo v.10.10.0.. All plots except 9f depict a brain sample. (1) Pre-gate used to reduce events for brain and blood samples. (2-4) Single cell gating. (5) Dead cell removal. (6) Debris exclusion. (7) Gating of CD45+ leukocytes. (8) Gating of CD3+ T cells. (9a) Gating of CD4+ T

© 2025. Gellrich J. et al. Published online at http://www.aginganddisease.org/EN/10.14336/AD.2025.0452

cells, CD8+ T cells and CD4-CD8-DNT. (9b) Gating of CD44-CD62L+ naïve T cells, CD44+CD62L+ central memory T cells and CD62L- effector/effector memory T cells. (9c-e) Gating of KLRG-1+, CD25+ and PD-1+ T cells. (9f, g) Gating of CD69+ T cells within brain samples (g) according to splenocyte FMO (f).

S4 Significantly higher number of GFP+ activated DNT in the cervical lymph nodes of AD mice at the age of 10-12 months.

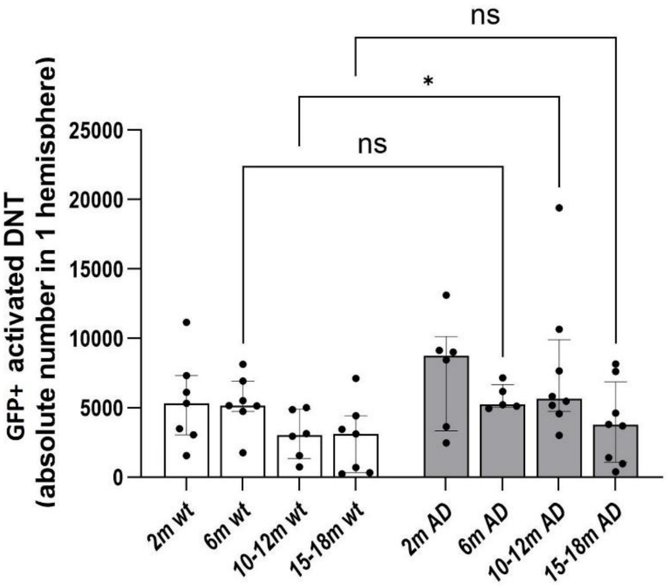

Supplementary Figure 2. Significantly higher number of GFP+ activated DNT in the cervical lymph nodes of of Alzheimer's dementia (AD) mice compared to healthy controls (wt) at the age of 10-12 months.
$n_{2m\ wt}=6$, $n_{6m\ wt}=7$, $n_{10\text{-}12m\ wt}=6$, $n_{15\text{-}18m\ wt}=7$, $n_{2m\ AD}=6$, $n_{6m\ AD}=5$, $n_{10\text{-}12m\ AD}=8$, $n_{15\text{-}18m\ AD}=8$.
Data information: Data are presented as median and IQR. Kruskal-Wallis test, post hoc Dunn's multiple comparison test for selected pairs (6m wt vs. 6m AD, 10-12m wt vs. 10-12m AD, 15-18m wt vs. 15-18m AD), *p =0.0488.

© 2025. Gellrich J. et al. Published online at http://www.aginganddisease.org/EN/10.14336/AD.2025.0452
